# Comparative EST transcript profiling of peach fruits under different post-harvest conditions reveals candidate genes associated with peach fruit quality

**DOI:** 10.1186/1471-2164-10-423

**Published:** 2009-09-10

**Authors:** Paula Vizoso, Lee A Meisel, Andrés Tittarelli, Mariano Latorre, Juan Saba, Rodrigo Caroca, Jonathan Maldonado, Veronica Cambiazo, Reinaldo Campos-Vargas, Mauricio Gonzalez, Ariel Orellana, Herman Silva

**Affiliations:** 1 Centro de Biotecnología Vegetal, Universidad Andrés Bello, Santiago, Chile; 2 Plant Functional Genomics & Bioinformatics Lab, Universidad Andres Bello, Santiago, Chile; 3 Millennium Nucleus in Plant Cell Biotechnology (MN-PCB), Santiago, Chile; 4 Laboratorio de Bioinformática y Expresión Génica, INTA-Universidad de Chile, Santiago, Chile; 5 Millennium Nucleus Center for Genomics of the Cell (CGC), Santiago, Chile; 6 Institute of Agricultural Research (INIA-La Platina), Santiago, Chile

## Abstract

**Background:**

Cold storage is used to inhibit peach fruit ripening during shipment to distant markets. However, this cold storage can negatively affect the quality of the fruit when it is ripened, resulting in disorders such as wooliness, browning or leathering. In order to understand the individual and combined biological effects that factors such as cold storage and ripening have on the fruit and fruit quality, we have taken a comparative EST transcript profiling approach to identify genes that are differentially expressed in response to these factors.

**Results:**

We sequenced 50,625 Expressed Sequence Tags (ESTs) from peach mesocarp *(Prunus persica *O'Henry variety) stored at four different postharvest conditions. A total of 10,830 Unigenes (4,169 contigs and 6,661 singletons) were formed by assembling these ESTs. Additionally, a collection of 614 full-length and 1,109 putative full-length cDNA clones within flanking loxP recombination sites was created.

Statistically analyzing the EST population, we have identified genes that are differentially expressed during ripening, in response to cold storage or the combined effects of cold storage and ripening. Pair-wise comparisons revealed 197 contigs with at least one significant difference in transcript abundance between at least two conditions. Gene expression profile analyses revealed that the contigs may be classified into 13 different clusters of gene expression patterns. These clusters include groups of contigs that increase or decrease transcript abundance during ripening, in response to cold or ripening plus cold.

**Conclusion:**

These analyses have enabled us to statistically identify novel genes and gene clusters that are differentially expressed in response to post-harvest factors such as long-term cold storage, ripening or a combination of these two factors. These differentially expressed genes reveal the complex biological processes that are associated with these factors, as well as a large number of putative gene families that may participate differentially in these processes. In particular, these analyzes suggest that woolly fruits lack the increased boost of metabolic processes necessary for ripening. Additionally, these results suggest that the mitochondria and plastids play a major role in these processes. The EST sequences and full-length cDNA clones developed in this work, combined with the large population of differentially expressed genes may serve as useful tools and markers that will enable the scientific community to better define the molecular processes that affect fruit quality in response to post-harvest conditions and the organelles that participate in these processes.

## Background

In order to improve fruit quality, it is important not only to take into account the quality of the fruit when it is harvested from the tree, but also post-harvest quality. After harvesting, fruits are packed and shipped to local and foreign markets. They remain on the shelf at supermarkets and/or farmers markets until the consumer purchases the fruits and finally consumes it. For this reason, the effects that shipping and storage have on fruit quality are very important. One of the most common methods to increase the post-harvest life of fruits is to store them in refrigeration, so that ripening is inhibited. However, prolonged cold storage of fruits such as peaches and nectarines can trigger a physiological disorder known as chilling injury or internal breakdown, which negatively affects fruit quality [1-3].

One of the most important problems associated with chilling injury is mealiness/woolliness. After prolonged cold storage, fruits produce a woolly phenotype when ripened. Woolly fruits lack juice and have a mealy texture, making them inedible and undesirable to consumers. This, in turn, leads to large economical losses for the fruit industry [4-6]. Chilling injuries such as wooliness are especially problematic for major counter-season fruit exporters such as Chile [4,7]. A better understanding of the molecular and cellular processes that lead to ripening, as well as woolliness, may provide future strategies to increase the shelf-life while minimizing the negative effects of cold-storage on fruit quality [8].

Wooliness occurs when fruits, stored between 0-8°C for prolonged periods of time, are transferred to conditions in which fruit ripening may occur. Under normal conditions, peach fruit ripening has been associated with changes in the expression of genes that code for cell wall degradation enzymes and the subsequent changes in enzymatic activity [9-11]. Various studies have provided correlative evidence that wooliness is associated with an imbalance between the activity of the cell wall-degrading enzymes, polygalacturonase (PG) and pectin methylesterase (PME) [9-11]. Additionally, by using macroarray analyses to compare the transcripts present in juicy fruits with those from wooliness, we have previously identified a number of genes that are differentially expressed in these fruits [12]. Ogundiwin et al, have also identified genes that are differentially expressed between juicy and woolly fruits using microarray analyses [13].

However, the susceptibility of peaches and nectarines to become woolly after prolonged cold-storage has been associated with particular peach and nectarine cultivars [3,14,15], as well as seasonal variation [3]. Cultivar specificity and seasonal variation suggest that multiple factors participate in developing a woolly phenotype in peach fruits [1,3]. Therefore, it is important to analyze not only the final end product (woolly fruits), but also the multiple factors that may be causing wooliness.

To identify the molecular mechanisms associated with developing wooliness, we have taken a global approach towards identifying genes that are differentially expressed in response to factors such as cold, ripening, or the combined effects of these two factors (cold + ripening), the latter of which results in the woolly peach phenotype.

We sequenced and statistically analyzed the abundance of Expressed Sequence Tags (ESTs) from peaches at four different post-harvest conditions, mimicking the stages of cold storage for fruit exportation. This statistical analysis has enabled us to identify target genes whose expression is affected by post-harvest factors such as long-term cold storage, ripening or the combined effects of these two factors. These analyses also reveal the different biological processes that are occurring under each post-harvest condition and the differences between these conditions. Additionally, we have generated a collection of full-length cDNA clones flanked by two loxP sites which may be useful in confirming the functionality of these gene products and the future annotation of the peach genome.

## Results and Discussion

In order to identify genes that are differentially expressed in response to factors such as long-term cold storage (C), genes that are differentially expressed during ripening (R), as well as the combined effects of these two factors (long-term cold storage and ripening), a 2 × 2 factorial design was used to generate four cDNA libraries that can be used for statistical comparative analyses [16].

These libraries represent the mesocarp tissue of O'Henry peaches (*Prunus persica*) under the following four distinct post-harvest conditions:

E1 = non-ripe; no long-term cold storage = R^-^, C^-^

E2 = ripe; no long-term cold storage = R^+^, C^-^

E3 = non-ripe; long-term cold storage = R^-^, C^+^

E4 = ripe: long-term cold storage = R^+^, C^+^

The maturity and physiological parameters of the fruits used to create these libraries are reported in Campos-Vargas et al [3]. These maturity and physiological parameters include firmness, percentage of total soluble solids, respiration rate and ethylene production [3].

**Figure 1:**
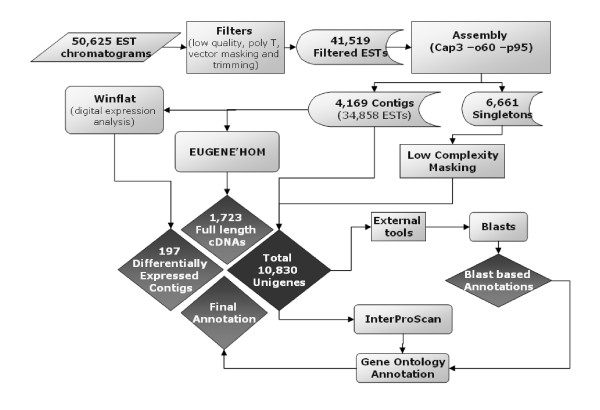
**Flowchart of the *in silico *EST analyses**. Chromatograms represent 5'EST end-sequences from four cDNA libraries of fruits with different post harvest treatments. Sequences were filtered, resulting in 41,519 ESTs that were used for assembling contigs. Assemblies generated 4,169 contigs and 6,661 singletons, resulting in a total of 10,830 Unigenes. The singletons were subsequently filtered for low complexity. The consensus sequence of each contig was further analyzed using EuGène'Hom to determine the number of full-length cDNAs. Using this analysis, 1,723 full-length cDNAs were identified (614 full-length cDNAs and 1,109 putative full-length cDNAs). Additionally, the number of ESTs in each contig was analyzed statistically to determine if the gene (s) that represent these contig are differentially expressed under the conditions analyzed [27].

5' end sequencing of clones from these libraries resulted in a total of 50,625 EST sequences. Assessment of these EST sequences revealed that a total of 41,519 ESTs are "good quality" sequences (average Phred Q>20 between bases 100 and 300 for each EST). Relatively equal numbers of "good quality" ESTs from each of the four post-harvest conditions were used for further analyses (Table 1, Figure 1, Additional File 1, Table S1).

**Table 1:** *Prunus persica *Unigene Set Statistics

	**# of EST sequences**	**Average length^b^**	**Maximum EST length^b^**	**Minimum EST length^b^**	**Assigned GO annotation^c^**
Total number of "good quality"^a ^ESTs	45,809	834	ND	ND	ND

ESTs without inverse ligation and PolyT	44,214	835	ND	ND	42,128

ESTs after trimmer	41,519	584	895	100	41,519

ESTs in contigs	34,858	589	895	100	34,858

ESTs in singletons	6,661	556	871	101	6,661

Contigs	4,169	861	3,082	109	4,169

Unigenes	10,830	673	3,082	101	10,830

^a ^Average Phred Q>20 between bases 100 and 300)

^b ^base pairs

^c ^Assigned GO annotations were derived from the annotation protocol

ND: not determined

As of May 2009, there were 79,023 *Prunus persica *EST sequences in NCBI, of which 32,497 ESTs correspond to sequences reported in this manuscript, but were publicly release to NCBI in 2006 (accession numbers: DY633390-DY654328, DW347789-DW359346). These 32,497 pre-release EST sequences also make up almost half of the sequences available in the ESTree database (75,404 ESTs) [17,18]. In addition to the sequences we have release previously, we are releasing an additional 9,022 EST sequences with the publication of this manuscript. This is a total of 41,519 ESTs which are being reported for the first time in this manuscript.

Assembling these 41,519 ESTs using CAP3 95/60 resulted in the identification of 10,830 Unigenes (4,169 contigs and 6,661 singletons). EST distribution in the contigs is presented in Additional File 1, Table S2. BLASTn and BLASTx analyses revealed that 98.9% of the contigs shared homology to sequences in public databases, whereas the remaining 47 contigs and 656 singletons showed no significant homology to the sequences in the public databases. Interestingly, although there is a high level of homology between the consensus sequences of our Unigenes and the sequences in public databases, 3,786 of our Unigenes contain novel sequence information, not present in public databases such as NCBI, ESTree, PlantTA, GDR and ChillPeach [13,17-21].

BLAST, Interproscan and BLAST2GO analyses were used to annotate the Unigenes and ESTs [22-26]. Gene Ontology (GO) annotations were assigned to the ESTs and contigs. Detailed information associated with the ESTs, contig consensus sequences and their associated annotations are available to browse or download at [27].

The length of the contig consensus sequences ranged from 0.1 to 3.0 kb, with an average length of 0.9 kb (Table 1). This large size detected in the consensus sequences suggests that some of these cDNA clones may be full-length. Analyses of the contig consensus sequences with EuGène'Hom [28] enabled us to identify the components of a full-length cDNA (5' UTR, ORF and 3' UTR). These analyses identified 614 full-length cDNAs, ranging in size from 0.4 to 2.7 kb (Table 2). Additionally, 1,109 contigs represent putative full-length cDNAs, in which the 5' UTR and ORF regions were detected. Since we performed 5'-end sequencing on these putative full-length cDNAs clones, they should contain the 3' UTR. Further 3' sequencing of these clones will confirm the full-length clones.

**Table 2:** Full-length cDNA sequences identified with EuGène'Hom.

**Category**	**Consensus Sequence Structure^a^**	**Average Length^b^**	**Maximum Length^b^**	**Minimum Length^b^**	**# Contigs**
1	**5' UTR - ORF - 3'UTR**	970	2,715	376	**614**

2	**5' UTR - ORF**	832	2,579	388	**1,109**

3	**5' UTR**	705	1,670	384	**115**

4	**ORF - 3'UTR**	900	2,670	241	**782**

5	**ORF**	834	3,082	149	**1,117**

6	**3'UTR**	709	1,334	109	**220**

7	**NA^c^**	936	2,323	525	**212**

	**Total**				**4,169**

^a ^The structure of the contig consensus sequence were analyzed using EuGène'Hom [21]. The results of this analysis were divided into 7 categories, 1: Full-length (5' UTR - ORF - 3'UTR; 2: Putative Full-length (5' UTR - ORF); 3: only 5' UTR; 4: only ORF - 3'UTR; 5: only ORF; 6: only 3'UTR; 7: none of the previous mentioned structures.

^b ^base pairs

^c ^NA: No identifiable structures

A summary of the results from this EuGène'Hom analysis is presented in Table 2. The clones of these full-length cDNAs have been identified by correlating the ESTs that are at the 5' end of the consensus sequences with the clones that these ESTs represent. Correlations between the insert size of many of these clones and the size of the contig consensus sequence suggests that the EuGène'Hom predictions are accurate (data not shown). Since our cDNA libraries were made in Clontech's pDNR-1r vector, these full-length and putative full-length cDNAs have flanking loxP recombination sites on both sites of the cDNAs [29,30].

### Differential expression of genes in each post-harvest stage

To estimate the distribution of ESTs from individual contigs among four cDNA libraries, a two-dimensional hierarchical clustering using pair-wise average-linkage cluster analysis [31] was applied. To ensure that contigs made up of a low number of ESTs are not unrepresented in the cluster analyses, the number of ESTs in each library was normalized proportionally to the total number of ESTs in their corresponding contig. The hierarchical clustering of the normalized contigs (Additional File 1, Figure S1), revealed that the distribution of ESTs in E1 (R^-^, C^-^) and E3 (R^-^, C^+^) are more similar to each other when compared to E2 (R^+^, C^-^) or E4 (R^+^, C^+^). Similarly, E2 (R^+^, C^-^) and E4 (R^+^, C^+^) are more similar to each other when compared to E1 (R^-^, C^-^) or E3 (R^-^, C^+^).

Relative gene expression can be determined by statistically comparing the number of ESTs from a gene between different libraries or different genes in the same library [32]. Pair-wise comparisons of the number of ESTs from each post-harvest condition, revealed differentially expressed genes (p < 0.01). Based on this statistical analysis, 197 contigs with at least one significant difference in gene expression were detected between the different post-harvest conditions. A total of 30 genes increase expression in ripe, juicy fruits (E2: R^+^, C^-^) when compared to non-ripe fruits (E1: R^-^, C^-^), whereas 39 genes are reduced (Additional File 2, Table S4). Long-term cold stored fruits (E3: R^-^, C^+^) increases the expression of 56 genes and decreases the expression of 45 genes when compared to fruits that have not undergone this long-term storage (E1: R^-^, C^- ^; Additional File 2, Table S5). Fruits that are ripened following long-term cold storage and are woolly (E4: R^+^, C^+^) have an increased expression of 36 genes and a decreased expression of 68 genes when compared to unripe fruits that have undergone long-term storage (E3: R^-^, C^+^; Additional File 2, Table S6). A total of 9 genes have a statistically significant increase in expression in woolly ripened long-term cold stored fruits (E4: R^+^, C^+^) when compared to juicy ripened fruits that have not been stored in the cold (E2: R^+^, C^-^), whereas 8 genes are reduced (Additional File 2, Table S7).

**Figure 2:**
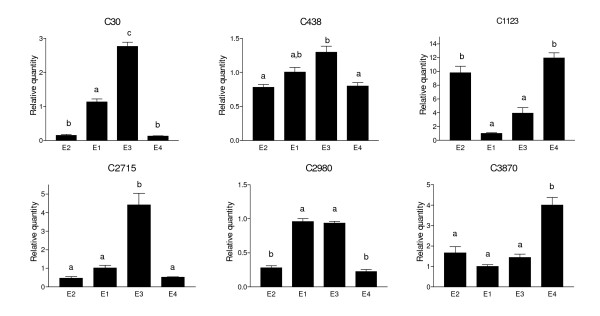
**Relative quantity of transcript levels in response to long-term cold storage, ripening, as well as the combination of these two factors**. The qPCR data was obtained by analyzing the transcript level of contigs 30, 438, 1123, 2715, 2980 and 3870) under four post-harvest conditions, normalized against a gene which did not demonstrate significant variations in transcript levels (Dehydrogenase/GMP reductase, contig 2766). Graphed qPCR data represents the mean transcript level of three or four individual fruits ± the standard error. E1 = non-ripe; no long-term cold storage = R^-^, C^-^; E2 = ripe; no long-term cold storage = R^+^, C^-^; E3 = non-ripe; long-term cold storage = R^-^, C^+^; E4 = ripe: long-term cold storage = R^+^, C^+^. contig30: Wcor; contig438: dormancy-associated protein (DRM1); contig1123: polygalacturonase; contig2715: luminal binding protein 1 (BIP-1); contig 2980: temperature-induced lipocalin (TIL) and contig 3870: lipoxygenase.

This *in silico *analysis was validated by performing qPCR analyses on several of the contigs that showed differential gene expression levels based upon the pair-wise *in silico *analyses (Figure 2, Additional File 2 Tables S4-S7). Figure 2 reveals differential gene expression confirmed by performing qPCR analyses of the following contigs under the four post-harvest conditions: Wcor (contig30), dormancy-associated protein (DRM1; contig438), polygalacturonase (contig 1123), luminal binding protein 1 (BIP-1; contig2715), temperature-induced lipocalin (TIL) (contig 2980), and lipoxygenase (contig 3870) (Figure 2). This comparative analysis revealed that the *in silico *and qPCR assays yield similar results for these genes, under the conditions analyzed.

### Ripening related genes

The pair-wise comparison of EST abundance of our contigs between two stages, revealed a significant increase in the transcript levels of 30 genes in these ripe fruits (E2: R^+^, C^-^) when compared to the unripe fruits (E1: R^-^, C^-^), and a reduction in the transcript levels of 39 genes (Additional File 2, Table S4). It is important to note that the fruits reported by Campos-Vargas et al [3] are the same fruits that were used for constructing our libraries. As reported by Campos-Vargas et al [3], ripe fruits were less firm than unripe fruits (7.9 N versus 72.5 N, respectively), but had a higher respiration rate (110.8 versus 28.8 mL CO_2 _kg^1 ^h^1^, respectively) and ethylene production (4.7 versus 1.6 μL C_2_H_4_kg^1^h^1^) [3,33-38].

The annotations of these differentially expressed transcripts reveal genes that participate in the physiological changes detected between ripe and unripe fruits. For example, 5 polygalacturonase transcripts (contigs1123, 1507, 1745, 3321, and 3904) are accumulated higher in ripe fruits than unripe fruits (Additional File 2, Table S4), which may lead to the changes in fruit firmness. These contigs share high identity (97-99%) with the polygalacturonase that was identified in microarray analyses of ripening fruits [11,39]. These results are consistent with the increase of endopolygalacturonase enzymatic activity in ripened peach fruits which has been reported previously [40,41].

In addition to the increased transcript accumulation of polygalacturonases, we detected a decreased expression of pectate lyase (contig 3683) and pectin methylesterase inhibitor genes (contigs 59 and 3598) (Additional File 2, Table S4). Trainotti et al [11] have detected increases in pectate lyase transcripts during the S4I phase of peach fruit ripening (climateric fruits that have not undergone softening, 115 days post-fertilization) and then decreases in the S4II phase (climateric fruits that have softened, 120-125 days post-fertilization). Our pair-wise comparisons of ESTs between ripe and unripe fruits detect a decrease in pectate lyase in ripe fruits when compared to unripe fruits (Additional File 2, Table S4). This decrease in pectate lyase gene expression as well as the reduction in firmness of the ripened fruits [3] suggests that our E2 stage is similar to the S4II stage described by Trainotti et al [11].

In addition to cell wall modifying enzymes, we have detected increased expression of genes associated with ethylene biosynthesis such as S-Adenosyl Methionine Synthetase (SAM Synthetase, contig 3949) and 1-Aminocyclopropane-1-Carboxylate Oxidase (ACC oxidase, contig 1901) (Additional File 2, Table S4). The increase in expression of this ACC oxidase (contig 1901) in ripe fruits has also been detected by Trainotti et al [39], whereas the SAM synthetase was not. The differential expression of these genes may also explain the increase in ethylene production detected in the ripe (E2: R^+^, C^-^) fruits.

As mentioned earlier, we have also detected an increased respiration in ripe fruits when compared to unripe fruits [3]. The pair-wise comparison reveals increased expression in several genes associated with glycolysis which may lead to this increase in respiration (Additional File 2, Table S4). These genes include Fructose-bisphosphate aldolase (contig 1358), glyceraldehyde 2-phosphate dehydrogenase (contig 1653) and pyruvate decarboxylase (contigs 1054 and 2749).

### Cold-storage related genes

Changes in firmness and respiration rate were also seen when unripe fruits stored at 4°C (E3: R^-^, C^+^) were compared to unripe fruits prior to storage (E1: R^-^, C^-^; firmness: 43.2 versus 72.5 N; Respiration rate: 94.3 versus 28.8 CO_2 _kg^1 ^h^1^, respectively) [3]. The pair-wise analyses reveal 56 genes that increase expression and 45 genes that decrease expression as a result of this long-term cold storage (Additional File 2, Table S5). Several of these genes are associated with cell wall organization and, therefore, may be associated with the changes detected in firmness. Genes that increase include: polygalacturonase inhibiting protein 1 (contig 2988), pectin methylesterase (contig 2877), basic endochitinase (contig 2131), and endopolygalacturonases (contigs 1507 and 1745). Genes that decrease include: expansin (contig 1200), invertases/pectin methylesterase inhibitors (contigs 59, 1945 and 3598), and pectate lyase (contig 3683). Among these cell wall associated contigs, only the basic endochitinase (contig 2131) has been reported previously to have altered expression in long-term cold stored fruits [13].

In addition to the altered expression of cell wall modifying proteins, we have also detected increased expression of glutathione peroxidase (contig 2514; Additional File 2, Table S5). Glutathione peroxidase is an oxidative stress responsive gene, which suggests that the fruits that have been stored for prolonged periods of time have an increase in oxidative stress, possibly associated with the increased respiration rates. Oxidative stress has been demonstrated in many species to be associated with chilling injury [42-45].

Among the genes differentially expressed between fruits that have undergone prolonged cold storage with those that have not, seven of these correspond to the genes that Ogundiwin et al [13] have also identified as having altered expression following cold storage (contigs 1546, 1693, 2131, 2311, 2495, 2957 and 2988). The remaining 94 contigs that are differentially expressed between unripe fruits stored at 4°C (E3: R^-^, C^+^) and unripe fruits prior to storage (E1: R^-^, C^-^) are novel and may participate in the adaptation and/or stress responses associated with long-term cold storage of these fruits.

### Wooliness response genes

When fruits that have been stored for long periods of time in cold storage (E3: R^-^, C^+^) are removed from this storage and ripened (E4: R^+^, C^+^), there is a significant decrease in firmness (43.2 versus 6.8 N, respectively), a significant increase in ethylene production (1.0 versus 18.1 μL C_2_H_4_kg^1^h^1^, respectively) and the fruits have a woolly phenotype [3]. The pair-wise analyses reveal an increased expression of 36 genes and a decreased expression of 68 genes in fruits that are ripened following long-term cold storage and are woolly (E4: R^+^, C^+^) in comparison to unripe fruits that have undergone long-term storage but are unripe (E3: R^-^, C^+^; Additional File 2, Table S6). Additionally, these analyses reveal an increased expression of 9 genes and decreased expression of 8 genes when woolly fruits are compared to juicy fruits, (E4: R^+^, C^+ ^and E2: R^+^, C^-^, respectively; Additional File 2, Table S7).

The changes in firmness and the wooliness phenotype have been reported previously to be correlated with modifications in the levels of cell wall modifying enzymes [1,9,40,41,46-49]. As mentioned earlier, our pair-wise analyses have identified five polygalacturonase transcripts with increased accumulation in ripe fruits when compared to unripe fruits (contigs 1123, 1507, 1745, 3321 and 3904; Additional File 2, Table S4-7). Of these five transcripts, two (contigs 1507 and 1745) have an increased accumulation following long-term cold storage. Ripening of the cold stored fruits produces an increased accumulation of four of these polygalacturonase transcripts (contigs 1507, 1745, 3321 and 3904). However, when comparing juicy fruits with woolly fruits, there is less accumulation of three of these transcripts (contigs 1123, 1507 and 1745) in woolly fruits. These results suggest that in woolly peaches there are diverse polygalacturonase transcripts which are unable to accumulate to the levels normally present in ripe juicy fruits. These results are complementary to several studies that have linked polygalacturonase activity with wooliness [1,9-12,39-41,46-50]. Interestingly, Peace et al [51] have also identified an endopolygalacturonase that co-localizes with a major QTL affecting both mealiness and bleeding. The large number of different polygalacturonase transcripts may be a result of alternative splicing or a recent gene duplication resulting in a highly conserved polygalacturonase gene family. The completion of the peach genome sequence will help to clarify whether these transcripts are a result of multiple genes or alternative splicing.

In addition to the changes in the accumulation of polygalacturonase transcripts, we have also identified a polygalacturonase inhibiting protein that has an inverse expression when compared to polygalacturonase in fruits that are ripened following a long-term cold storage and have a woolly phenotype. That is, there is a reduced expression of the polygalacturonase inhibiting protein in woolly fruits (E4: R^+^, C^+^) when compared to unripe fruits that have undergone long-term cold storage (E3: R^-^, C^+^). This reduction in the expression of the polygalacturonase inhibiting protein may lead to an increase in polygalacturonase activity and subsequent reduction in the fruit firmness. A similar pattern of reduced expression in fruits that are ripened following a long-term cold storage is also seen for another cell wall modifying enzyme, endochitinase (contig 2131).

A decrease in pectate lyase expression (contig 3683) was detected when comparing ripen fruits E2 (R^+^, C^-^) with unripe fruits E1 (R^-^, C^-^). However, a decrease in expression of this contig was also detected in fruits stored for prolonged periods of time in the cold (E3: R^-^, C^+ ^versus E1: R^-^, C^-^), suggesting that this decrease may be similar to the decrease detected by Trainotti et al [11] in the S4II stage (climateric fruits that have softened, 120-125 days post-fertilization). Interestingly, the expression levels of this pectate lyase increased in woolly ripened long-term cold stored fruits (E4: R^+^, C^+^) when compared to long-term cold stored fruits (E3: R^-^, C^+^). We have previously reported a decreased transcript level of pectate lyase when woolly fruits were compared to juicy fruits by macroarray analyses [12]. One explanation for this is that the cold-storage may have reduced the pectate lyase transcript to a point that despite the increased expression of pectate lyase in woolly ripened long-term cold stored fruits (E4: R^+^, C^+^) when compared to long-term cold stored fruits (E3: R^-^, C^+^), the transcript levels in woolly fruits (E4: R^+^, C^+^) are still lower than the levels detected in juicy fruits (E2: R^+^, C^-^). Alternatively, there may be a family of pectate lyase genes that are differentially expressed in the fruits, postharvest. It has been reported that in numerous species, pectate lyases are encoded by a large gene family [52]. We have identified three putative pectate lyases among our Unigenes (contigs 328, 1989 and 3683), suggesting that in peaches there is a pectate lyase gene family.

Trainotti et al [11] have demonstrated that three different expansin genes are expressed differentially during ripening. Two of these expansin genes are up-regulated and one is down regulated in the S3/S4 transition [11]. We have identified seven contigs that are annotated as expansin or expansin family proteins (contigs 618, 1200, 1520, 1680, 2060, 2212 and 2420). The expansin genes analyzed by Trainotti et al [11] correspond to contigs 3786 and 1951 in our unigene sets. Interestingly, we have identified a novel expansin (contig 2212) that has increased transcript levels in woolly fruits when compared to juicy fruits. Since there are higher levels of the transcript for this contig in ripe, juicy fruits (E2: R^+^, C^-^) in comparison to unripe fruits that have been stored for a long period of time (E3: R^-^, C^+^), the increase in expansin levels in woolly fruits (E4: R^+^, C^+^) is occurring during the ripening process by a factor that cold storage has affected, and is not due to an increase in expansin levels in cold-stored fruits.

Trainotti et al [11] have also seen an increased expression of pectin methylesterase in S4I (climateric fruits that have not undergone softening, 115 days post-fertilization) and S4II ripened fruits (climateric fruits that have softened, 120-125 days post-fertilization). Our pair-wise comparison has detected an increased pectin methylesterase-like protein transcript (contig 2877) in woolly fruits when compared to juicy fruits. Increased transcripts that correspond to this contig were detected when comparing long-term cold storage fruits (E3: R^-^, C^-^) with fruits from packing (E1: R^-^, C^-^). This suggests that the increase in this pectin methylesterase gene was induced by cold storage and remained elevated following ripening and the development of the woolly phenotype.

In addition to the changes in the expression of pectin methylesterase, we have detected eight pectin methylesterase inhibitor proteins in our Unigenes (contigs 59, 422, 741, 1062, 1945, 2574, 3374 and 3598). Pair-wise analyses detected differential expression of three of these contigs (contigs 59, 1945 and 3598). Two of these contigs (contigs 59 and 3598) have similar patterns of differential expression under different post-harvest conditions. Both of these contigs have a reduced expression in ripe fruits with and without long-term cold storage (Additional File 2, Table S4-7). Contig 1945 also has a reduced expression in ripe juicy fruits. However, this contig has an increased expression in woolly ripened long-term cold stored fruits (E4: R^+^, C^+^) when compared to unripe fruits that have undergone long-term storage (E3: R^-^, C^+^; Additional File 2, Table S6). In the model plant Arabidopsis, there is a family of pectin methylesterase inhibitors and invertases [53]. In vitro studies of pectin methylesterase inhibitors have been shown to have specific targets [53]. Therefore, the differential expression between the different contigs that code for pectin methylesterase inhibitors may have different targets. However, it is important to mention that these different contigs may be the product of different transcripts from a single locus, rather than a gene family. Future mapping of these ESTs to the peach physical map will help to clarify this possibility [54].

As mentioned earlier ethylene production has been shown to participate in peach fruit ripening [10,39]. We have detected an ACC oxidase gene (contig 1901) whose expression is increased in ripe fruits when compared to unripe fruits. However, there is a reduction in ACC oxidase (contig 1901) expression in woolly fruits (E4: R^+^, C^+^) when compared to juicy fruits (E2: R^+^, C^-^). This reduced expression of ACC oxidase may be associated with the wooliness phenotype. However, the ethylene production in the woolly fruits was higher than juicy fruits [3], suggesting that there are other genes associated with ethylene production that may also be altered.

We have also identified a number of genes associated with membrane formation and stress related genes that are differentially expressed under the different postharvest conditions analyzed. A large number of the genes that we have identified as being differentially expressed in response to cold, ripening or a combination of these two stimuli, have not been reported previously. Therefore, the large number of genes identified as having altered expression in response to the individual or combined effects of ripening and long-term cold storage, demonstrates the complexity of the processes that are occurring in peach fruits post harvest and the differential expression of gene families. Further analyses of the expression of these genes and the factors that cause this differential expression may provide interesting clues about the mechanisms associated with wooliness.

### Identification of genes that are co-expressed under different post-harvest conditions

**Figure 3:**
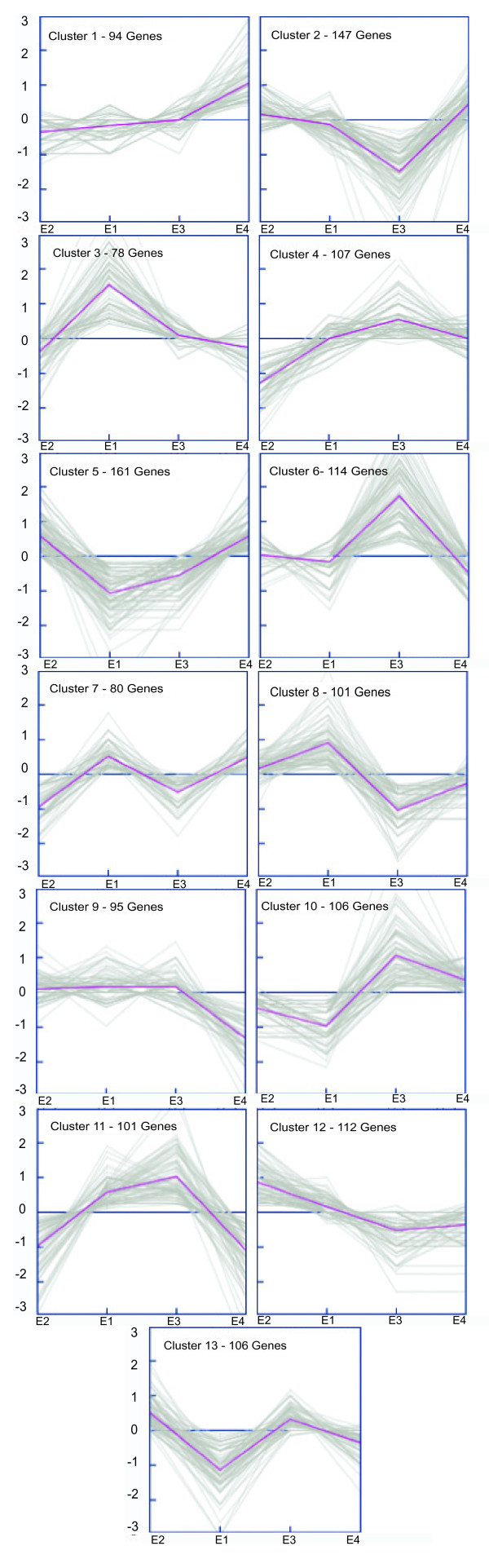
**Expression profiles of clusters of genes with similar expression patterns**. K-means clustering was performed with 1,402 normalized Unigenes with five or more ESTs. Relative expression levels (log2 ratios) of these clusters of genes are shown at different post-harvest conditions, the mean expression values are represented by the magenta lines. Genes were grouped into 13 clusters with distinct expression profiles. The optimal number of clusters was determined by Figure of Merit Analyses (Supplementary Figure S2). The expression levels for ripe juicy fruits that have not under-gone long-term cold storage, E2 (R^+^, C^-^); non-ripe; non-long-term cold storage fruits, E1 (R^-^, C^-^); and non-ripe and ripe fruits that have undergone long-term cold storage, E3 (R^-^, C^+^) and E4 (R^+^, C^+^), respectively, appear on the graphs. The total number of genes that make up each cluster is represented in the upper left hand corner of each cluster.

To identify clusters of genes that are co-expressed under different post-harvest conditions, a *k*-means analysis was performed to identify groups of genes with similar expression profiles. *K*-means clustering was performed using 13 different clusters with a maximum of 50 iterations, each containing between 78 and 161 genes (Figure 3). The optimal number of clusters was determined to be 13 by figure of merit analyses (Additional File 1, Figure S2).

**Figure 4:**
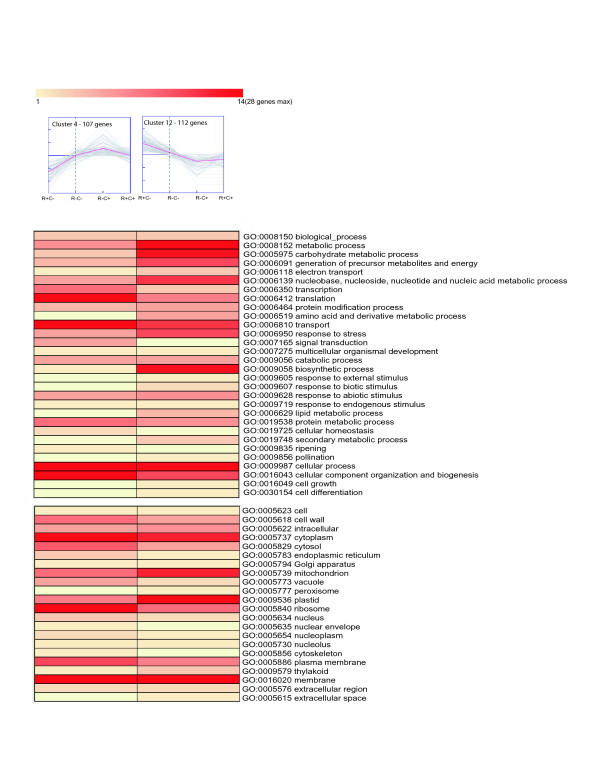
**Gene clusters with inverse expression profiles exclusively in ripened fruits**. Cluster 4 (left) contains 107 genes that have reduced expression exclusively in juicy, mature fruits (E2: R^+^, C^-^), whereas cluster 12 (right) contains 112 genes with increased expression exclusively in these same fruits. The Gene Ontology annotations of the genes in these clusters are represented below each cluster. The number of genes associated with each GO term is represented by the color of the bars, as depicted in the scale presented in this figure. The dashed line indicates non-ripe fruits, E1 (R^-^, C^-^). To the left of the dashed line is the expression levels for ripe, juicy fruits, E2 (R^+^, C^-^). To the right of the dashed line are E3 (R^-^, C^+^) and E4 (R^+^, C^+^) fruits, non-ripe and ripe fruits that have undergone long-term cold storage, respectively. E4 (R^+^, C^+^) fruits are wooly. GO annotations correspond to the parental plant GO Slim terms.

**Figure 5:**
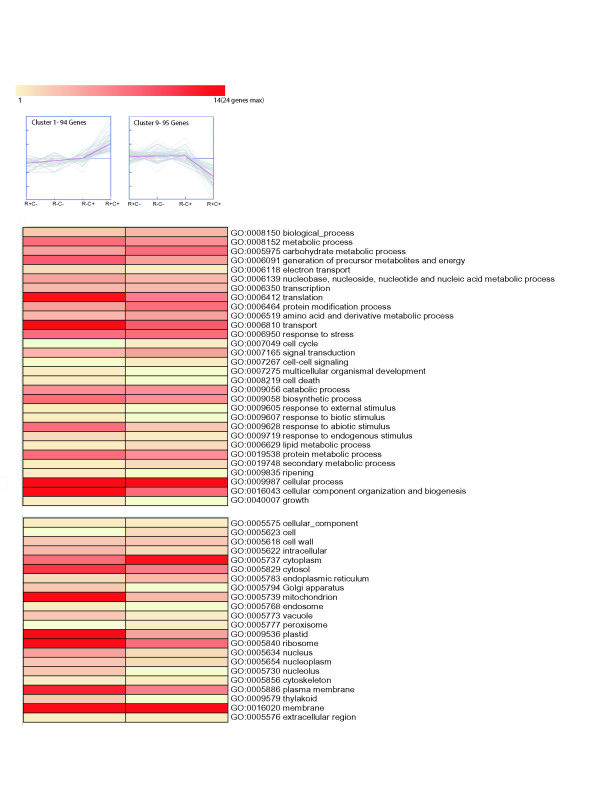
**Gene clusters with inverse expression profiles exclusively in woolly fruits**. Cluster 1 (left) contains 94 genes that have increased expression exclusively in fruits that are wooly (E4: R^+^, C^+^), whereas cluster 9 (right) contains 95 genes with decreased expression exclusively in these same fruits. The Gene Ontology annotations of the genes in these clusters are represented below each cluster. The number of genes associated with each GO term is represented by the color of the bars, as depicted in the scale presented in this figure. The dashed line indicates non-ripe fruits, E1 (R^-^, C^-^). To the left of the dashed line is the expression levels for ripe, juicy fruits, E2 (R^+^, C^-^). To the right of the dashed line are E3 (R^-^, C^+^) and E4 (R^+^, C^+^) fruits, non-ripe and ripe fruits that have undergone long-term cold storage, respectively. E4 (R^+^, C^+^) fruits are wooly. Annotations correspond to the parental plant GO Slim terms.

**Figure 6:**
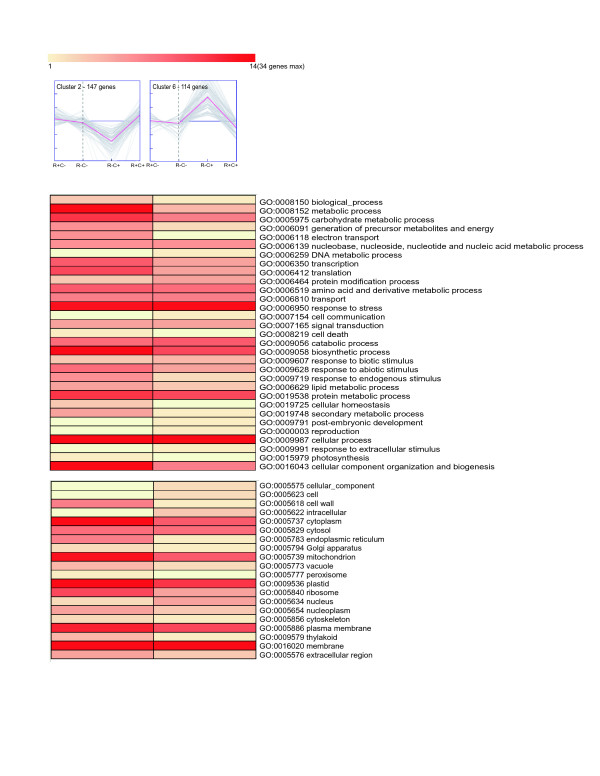
**Gene clusters with inverse expression profiles exclusively in fruits that have undergone long-term cold storage**. Cluster 2 (left) contains 147 genes that have reduced expression exclusively in fruits that have undergone long-term cold storage (E3: R^-^, C^+^), whereas cluster 6 (right) contains 114 genes with increased expression exclusively in these same fruits. The Gene Ontology annotations of the genes in these clusters are represented below each cluster. The number of genes associated with each GO term is represented by the color of the bars, as depicted in the scale presented in this figure. The dashed line indicates non-ripe fruits, E1 (R^-^, C^-^). To the left of the dashed line is the expression levels for ripe, juicy fruits, E2 (R^+^, C^-^). To the right of the dashed line are E3 (R^-^, C^+^) and E4 (R^+^, C^+^) fruits, non-ripe and ripe fruits that have undergone long-term cold storage, respectively. E4 (R^+^, C^+^) fruits are wooly. GO annotations correspond to the parental plant GO Slim terms.

Groups of clusters that correlate the gene expression profiles with the different post-harvest conditions are clearly visible when these groups are analyzed and compared (Figure 4, 5, and 6). One group (Figure 4) is represented by two clusters of genes (Clusters 4 and 12) whose expression profiles are inversely related in juicy, ripe fruits (E2: R^+^, C^-^), when compared to the other post harvest stages. Cluster 4 contains 107 genes with reduced expression exclusively in juicy, ripe fruits (E2: R^+^, C^-^), whereas cluster 12 contains 112 genes with increased expression exclusively in these same fruits. It is important to note this comparison is not a pair-wise comparison, rather a comparison between all four post-harvest conditions (E1: R^-^, C^-^; E2: R^+^, C^-^; E3: R^-^, C^+^; E4: R^+^, C^+^). Clusters 4 and 12 reveal a decrease and increase in gene expression in juicy fruits, respectively, which is not present in woolly fruits. Genes that are differentially expressed in juicy fruits and not woolly fruits are genes that may be participating in the woolly phenotype. Analyses of these sequences and their corresponding Gene Ontology annotations reveal that ripe peaches have an increase in metabolic processes, especially processes associated with the metabolism of carbohydrates, nucleic acids, amino acids, lipids and secondary metabolites (Figure 4). In contrast, there is a decrease in translation, transport, and signal transduction processes. According to the GO annotations, it appears as though there is an increase in the expression of genes that are associated with mitochondria and plastids, as well as a decrease in those associated with ribosomes and the plasma membrane. Many of the metabolic process described earlier are carried out in the mitochondria and plastids, supporting this trend. The decrease in translation also correlates with the decrease in processes associated with the ribosome. These analyses suggest that woolly fruits lack the increased boost of metabolic processes necessary for ripening, such as the expression of cell wall modifying enzymes as well as the production of sugars and aromatic secondary metabolites. The changes in expression of cell wall modifying enzymes is consistent with what has been reported previously [1,9-13,39,48,51,55,56]. However, these cluster analyses reveal the organelles that may actively participate in these processes as well as a large number of genes and gene families that should be analyzed for their participation in the altered metabolic processes present in woolly fruits.

In addition to the absence of the modified gene expression patterns seen in clusters 4 and 12, there is another pair of clusters that represent the opposite tendency. Clusters 1 and 9 (Figure 5) represent genes that change their expression exclusively in woolly fruits E4 (R^+^, C^+^). Cluster 1 contains 94 genes with increased expression exclusively in woolly fruits, whereas cluster 9 contains 95 genes with decreased expression exclusively in woolly fruits. In these clusters, there is a notable increase in the expression of genes associated with processes such as translation, transport, response to abiotic stress and cellular organization and biogenesis. Interestingly, the combined results of clusters 4 and 1 (Figures 4 and 5) demonstrate that woolly fruits have lost the reduced expression of genes associated with transcription and translation. Additionally, following abiotic stress conditions such as long-term cold storage, it is not surprising to see increased expression of abiotic stress related genes in woolly fruits. However, it should be noted that this class of abiotic stress related genes are distinct from those identified exclusively in the cold (Figure 6). As was seen in clusters 4 and 12, many of the differentially expressed genes that are differentially expressed exclusively in woolly fruits appear to be associated with the mitochondria, plastids and ribosomes. The participation of the mitochondria and plastids also correlate well with the increase in respiration that was detected in these fruits [3]. The plasma membrane is also participating, possibly associated with abiotic stress responses such as cold and/or desiccation stresses.

The identification of genes that are associated with mitochondrial and plastid processes, as well as the differential expression of genes such as glutathione peroxidase, suggests that oxidative stress and metabolic imbalances may play a major role in wooliness. Interestingly, recent publications have presented physiological analyses that support this hypothesis [57-59].

Yet another group represents genes that are differentially expressed exclusively in unripe fruits that have undergone long-term cold storage E3 (R^-^, C^+^; Figure 6). This group is represented by clusters 2 and 6. Cluster 2 contains 147 genes with reduced expression exclusively in non-ripe fruits that have undergone a long-term cold storage (E3: R^-^, C^+^), whereas cluster 6 contains 114 genes with increased expression exclusively in these same fruits. Surprisingly, only 4 of the genes, present in cluster 6 correspond to genes identified as being differentially expressed by Ogundiwin et al [13]. Differentially expressed genes, identified by Ogundiwin et al [13], appear in clusters 6, 7, 11 and 12. Therefore, we have identified a very large number of novel genes that are associated specifically with the unripe fruits that have undergone long-term cold storage. These genes are associated with a large number of biological processes in multiple regions of the cell. By further analyzing the function of these genes we may be able to better understand the specific modifications that have occurred in the fruit under long-term cold storage that ultimately results in the modification in the ripening process that result in woolly fruits.

## Conclusion

By sequencing a large number of ESTs from cDNA libraries representing peach mesocarp from four different post-harvest conditions, we have begun to identify the individual and combined effects that long-term cold storage (C) and ripening (R) have on the transcripts in this tissue.

10,830 Unigenes (4,169 contigs and 6,661 singletons) were formed by assembling a total of 41,519 ESTs. These EST sequences have been deposited in Genbank and the sequence information associated with the Unigenes is available at . Additionally, since the libraries that were used in this sequencing effort were enriched for large inserts and are cloned within two flanking lox-P sites in the pDNR-1r vector (Clontech), we have a collection of full-length clones which may be easily recombined with vectors that contain single loxP sites, for rapid cloning into other expression vectors and for functional analyses of these genes.

In addition to the sequence information and full-length clones developed in this work, digital analyses of EST abundance in the four different post-harvest conditions has revealed a large number of candidate genes with fluctuating transcript levels in response to factors such as ripening, long-term cold storage and a combination of these two factors (ripening + long-term cold storage).

These analyses have enabled us to statistically identify novel genes and gene clusters that are differentially expressed in response to post-harvest factors such long-term cold storage, ripening and/or a combination of these two factors. These differentially expressed genes reveal the participation of specific metabolic processes that normally occur in ripen fruits that do not occur in woolly fruits, as well as novel processes that are occurring in the woolly fruits that are not normally present in ripen fruits.

These analyses also demonstrate the need to better identify and understand the specificity and expression of gene families and/or different transcripts from a single gene in post-harvest processes. Additionally, these analyses present data that suggests that wooliness is not only due to modifications of the cell wall, but may also include stress response pathways and oxidative damage.

The EST sequences and full-length cDNA clones developed in this work, combined with the large population of differentially expressed genes may serve as useful tools and markers that will enable the scientific community to better understand the molecular and cellular processes that affect fruit quality in response to post-harvest conditions and the large number of gene products that participate in these processes. By understanding these processes, this knowledge may be used in the future to improve post-harvest fruit quality.

## Methods

### cDNA library construction

*Prunus persica var. persica *(L.) Batch cv. O'Henry fruits were selected as the source of material for the construction of the cDNA libraries. Mesocarp tissue was collected from fruits at four different post-harvest conditions. The post-harvest conditions include: fruits processed in a packing plant (E1: R^-^C^-^: non-ripe; no long-term cold storage); packing followed by a shelf life at 20°C for 2-6 days (E2: R^+^C^-^: ripe; no long-term cold storage; juicy fruits); packing followed by cold storage at 4°C for 21 days (E3: R^-^C^+^: non-ripe; long-term cold storage) and packing followed by cold storage at 4°C for 21 days and shelf life at 20°C for 2-6 days (E4: R^+^C^+^: ripe; long-term cold storage; woolly fruits). In order to ensure that all fruits were similar in size and quality, fruits were selected in a commercial peach packing plant prior to postharvest treatment. The mesocarp tissue used for library construction corresponds to the fruits harvested in Year 1 (2003) and reported in Campos-Vargas et al [3]. The juice content (woolliness), maturity (ground color, firmness, total soluble solids) and physiological parameters (respiration rate and ethylene production) of the fruit tissue are described in Campos-Vargas et al (Year 1) [3].

We have previously reported the quality of these libraries (% Recombination and Clone Range) [60]. During the cDNA synthesis process, an adaptor containing an XhoI restriction site (5'-GAC TAG TTC TAG ATC GCG ACT CGAGCC-(T)_15_-3') was incorporated to the 3' end of the cDNA. cDNAs were enriched for large fragments and these fragments were subsequently cloned directionally into the pDNR-1r vector (Clontech) at the XhoI -SmaI sites. The clone codes uploaded in GenBank are PU1 for E1, PU2 for E2, PU3 for E3 and PU4 for E4.

### Sequencing and assembly

Arrayed cDNA clones from the four libraries were Sanger sequenced at the 5' end, using the M13 Forward primer. The EST data files received were used in our sequence analysis pipeline as shown in Figure 1. Sequence chromatograms were read with PHRED base calling software [61,62]. Only those ESTs that contained an average Phred Q > 20 between bases 100 and 300 were used for further analyses. Selected EST sequences were filtered in order to improve the quality of sequence assemble. These filters include: masking of bases that correspond to vector sequence, detection and filtering of reverse ligations or sequences with greater than 40 sequential Ts. Masked bases were subsequently removed. A 3' end quality filter was applied and sequences shorter than 100 bps were eliminated from further analyses. Sequences that passed these filters were assembled using CAP3 95/60 (-p 95 -d 60) [63].

### Annotation

Unigenes and ESTs were annotated based on accumulative bioinformatics evidence [64]. This evidence was obtained using BLAST [25] and InterProScan [26] against the following databases: GenBank (, Jan 2008 release); TAIR (, Jul 2007 release), and GDR (, March 2007 release). BLAST-based annotations were performed using BLASTn and BLASTx (BLOSUM62). InterProScan analyses integrate the PROSITE, PRINTS, Pfam, ProDom, SMART and TIGRFAMs databases [26]. All putative annotations with an E-value >10^-7 ^were selected and categorized according to the percentage of identity, length of coverage and the existence of gaps. Computational evidence associated with predicted gene structure and protein domains were also taken into consideration. This information was gathered and curate manually. Once validated, gene products were named according to the degree of similarity, structure and protein domains. To our knowledge, a standard nomenclature does not exist for peach annotations; therefore, we adapted our annotation protocol to include the prefixes and suffixes used by TAIR, TGR and RIKENs [65]. These prefixes and suffixes illustrate the degree of confidence based on computational evidence. The results of these analyses were stored in the JUICE data management system [27].

Functional classification of the Unigenes and ESTs were performed by assigning Gene Ontology annotation codes [66], based upon homology information gathered from the BLAST, BLAST2GO and InterProScan analyses [22-26].

Comparisons between ESTs and/or Unigenes were performed by mapping the GO annotations to their parent plant GO Slim terms using map2slim.pl script [67]. Only the matches with E-values less than 1e^-7 ^were included in the analysis. Unigenes were classified into multiple GO Slim Plant categories [68].

To find contaminating sequences, a BLASTn match with an alignment greater than 100 bp was used (≥ 90% and e-value ≤ than 10^-7^) [69]. EST sequences that met these criteria were not assigned GO annotations and were excluded from digital expression analyses since they are nearly identical to viral, fungal or bacterial sequences and, therefore, may represent contaminations.

Novel EST sequences were determined by performing BLASTn against the sequence information available in the public databases such as NCBI, ESTree, ChillPeach and PlantTA [13,17-19]. A sequence that does not contain novel sequence information is defined as those unigene consensus sequences that have ≥ 95% percent-length and ≥ 50% coverage with other published sequences. Sequences that did not meet these criteria are defined as sequences that contain novel sequence information.

### Digital gene expression profile

Gene expression profiles were analyzed statistically to determine if the Unigenes were differentially expressed under different post-harvest conditions using the Winflat program that submits the sequence data to a rigorous statistical analysis described by Audic and Claverie [32]. This approach allows differentially expressed genes (p < 0.01) to be identified from pair-wise cDNA library comparisons.

To identify groups of genes with similar expression profiles, coordinated gene expression analyses were performed on the peach EST data from the four cDNA libraries. Only contigs composed of at least five ESTs were used in making the expression profile matrix [31]. A hierarchical clustering method was applied to compare differential expression of these contigs. The number of ESTs in each post-harvest condition was normalized proportionally to the total number of ESTs in their corresponding contig. The displayed expression profile was normalized (log2). The green color indicates fewer and red indicates a greater number of ESTs than the norm for each contig [70].

To identify groups of co-expressed genes, we use the *k*-means method [71,72] with Pearson's correlation coefficient using the Genesis software tools [73] to cluster contigs that contain at least five ESTs. To determinate the optimal number of clusters, Figure of Merit (FOM) analyses were calculated to determine the quality of the clusters [74].

### qPCR analysis

Total RNA was prepared from E1, E2, E3 and E4 using the method described by Meisel et al [60]. cDNA was synthesized from 2.5 μg of total RNA using ImProm-IITM Reverse Transcription System kit (Promega) in 20 μL of reaction mixture. As a control, samples were spiked with 0.5 pg of Kanamycin RNA. 0.1 μL of the cDNA was added to 20 μL of PCR mixture containing each primer (0.2-0.5 μM), fast SYBR green master mix (2×) from Applied Biosystems. After heating at 95°C for 20 sec, PCR reactions proceeded through 45 cycles of 3 seconds at 95°C, 5 to 20 seconds at 60-62°C. The amount of the amplified DNA was monitored by fluorescence at the end of each cycle using Step One Real Time PCR System (Applied Biosystems). The primers used for qRT-PCR are described in Additional File 1, Table S3.

Quantification was based on the fact that the cycle threshold value (cycle number required to obtain a fluorescence signal above the background) correlates inversely with the log of the initial template concentration [75]. The relative abundance of the targeted mRNAs from several samples was determined from a standard curve that was constructed from a set of DNA dilutions from each one of the transcripts to be analyzed. To standardize the results, we used qRT-PCR for the dehydrogenase/GMP reductase (contig 2766), gene that under our experimental conditions does not show changes in transcript levels. The primers for dehydrogenase/GMP reductase are also described in Additional File 1, Table S3.

### Identification of full-length EST cDNA clones

The cDNA libraries were enriched for large fragments [60]. The consensus sequences of the Unigenes were analyzed with EuGeneHom [28], in order to identify the Unigenes that contained the components of a complete cDNA (5' UTR, ORF and 3' UTR) .

## Authors' contributions

PV annotated and curated the sequences and performed the k-means analyses. PV and AT performed the digital expression analyses. PV, ML, JS, LM and HS developed and implemented the assembly, annotation and bioinformatics analyses of the sequences. RC and PV performed the qPCR analyses. RC-V performed the postharvest treatment of the fruits; HS and LM conceived, supervised and participated in all analyses. PV, HS and LM drafted the manuscript. HS, VC, RC, MG, AO and LM supervised the Chilean Functional Genomics Consortium in Nectarines which provided the platform for this EST project workflow. All authors read and approved the manuscript.

## Supplementary Material

Additional file 1 **Supplementary Tables S1 - S3, Supplementary Figures S1 and S2**. This file contains additional information about the sequence analyses as well the primers used for qRT-PCR analyses. There are a total of three tables and figures. The titles of these tables and figures are as follows: Table S1 - Number of "Good Quality" ESTs sequenced from each post-harvest condition; Table S2 - Distribution of ESTs in Contigs; Table S3 - Primer sequences sets for qRT-PCR analyses of representative differentially expressed contigs; Figure S1 - The distribution of peach mesocarp derived ESTs under four different post-harvest conditions, using hierarchical clustering; Figure S2 - Figure of Merit Analysis. Click here for file

Additional file 2 **Supplementary Tables S4 - S7**. This file contains the contigs that digital expression analyses revealed are differentially expressed in different post-harvest conditions. The titles for these tables are as follows: Table S4 - Contigs that are differentially expressed between ripe peach fruits (E2: R+, C-) and unripe fruits (E1: R-, C-); Table S5 - Contigs that are differentially expressed between unripe peach fruits that have undergone long-term cold storage (E3: R-, C+) and unripe peach fruits that have not (E1: R-, C-); Table S6 - Contigs that are differentially expressed between ripen peach fruits that have undergone long-term cold storage (E4: R+, C+) and unripe peach fruits that have undergone long-term cold storage (E3: R-, C+); Table S7 - Contigs that are differentially expressed between ripen peach fruits that have undergone long-term cold storage (E4: R^+^, C^+^) and ripen peach fruits that have not undergone long-term cold storage (E2: R^+^, C^-^). Click here for file
