# Supplementary material for: Comparative EST transcript profiling of peach fruits under different post-harvest conditions reveals candidate genes associated with peach fruit quality
Source: BMC Genomics. 2009 Sep 10;10:423. doi: 10.1186/1471-2164-10-423 (PMC2748099; doi:10.1186/1471-2164-10-423)
Supplement: Additional file 1 — Supplementary Tables S1 - S3, Supplementary Figures S1 and S2. This file contains additional information about the sequence analyses as well the primers used for qRT-PCR analyses. There are a total of three tables and figures. The titles of these tables and figures are as follows: Table S1 - Number of "Good Quality" ESTs sequenced from each post-harvest condition; Table S2 - Distribution of ESTs in Contigs; Table S3 - Primer sequences sets for qRT-PCR analyses of representative differentially expressed contigs; Figure S1 - The distribution of peach mesocarp derived ESTs under four different post-harvest conditions, using hierarchical clustering; Figure S2 - Figure of Merit Analysis. [file 1471-2164-10-423-S1.doc]

**Additional File 1:**

**Supplementary Tables 1-3, Supplementary Figures 1-2.**

Table S1: Number of “Good Quality” ESTs sequenced from each post-harvest condition.

|  |  | | **# ESTs (% of total)** | | | | | | | |
| --- | --- | --- | --- | --- | --- | --- | --- | --- | --- | --- |
|  | **Total** | | **E1 (%)** | | **E2 (%)** | | **E3 (%)** | | **E4 (%)** | |
| ESTs | | 41,519 | | 10,449 (25.2) | | 10,154 (24.5) | | 10,573 (25.4) | | 10,343 (24.9) |
| Contigs | | 34,858 | | 8,896 (25.5) | | 8,515 (24.4) | | 8,919 (25.6) | | 8,528 (24.5) |
| Singletons | | 6,661 | | 1,553 (23.3) | | 1,639 (24.6) | | 1,654 (24.8) | | 1,815 (27.2) |

EST sequences where filtered using Phred quality Q<20 between bases 100 and 300 for each EST. The number of ESTs from each condition is presented. The number in parenthesis is the percentage of ESTs from each condition that makes up the total number of ESTs from all four conditions.

Table S2: Distribution of ESTs in Contigs.

| **<5 ESTs/Contig** | **≥5 ESTs/Contig** | | |
| --- | --- | --- | --- |
| 2,767 Contigs | 1,402 Contigs | | |
|  | **5<X≤10** | **10<x<100** | **≥100** |
|  | 837 Contigs | 532 Contigs | 33 Contigs |

The distribution of ESTs in the contigs was analyzed by determining how many ESTs were present in each contig. The numbers represent the number of contigs that meet the criteria displayed in the gray cells of the table.

**Table S3: Primer sequences sets for qRT-PCR analyses of representative differentially expressed contigs**

| **Code** | **Annotation** | **Forward primer (5' - 3')** | **Reverse primer (5' - 3')** |
| --- | --- | --- | --- |
| C3870 | lipoxygenase | 5' TGGCAAAGCCCACGTCTCGTCCAA 3' | 5' CAATCTCGATAAGCCGCACAACT 3' |
| C1123 | polygalacturonase | 5' GCATTGGAAGTCTAGGCAAGGAG 3' | 5' TTGTCGGGGCAATAATGTTGATCTA 3' |
| C30 | Wcor413 | 5’ TTCAACATCTTCAGGGGACAG 3’ | 5’CACCAATGCCCAAACAGG3´ |
| C438 | dormancy-associated protein | 5' GACAAGCCGCAGCCCAACTCT 3' | 5' TCCAAACCACCACCAAGGCAC 3' |
| C2715 | luminal binding protein 1 | 5' CAACCGCAGCCGCCATCGCATAT 3' | 5' CACGCTCAGCTTCCCTCCTCAAT 3' |
| C2980 | temperature-induced lipocalin | 5' CCAGCCTAGCAGGAATTACCTTT 3' | 5' ATTTGAACCACCAGATGCCCTTG 3' |
| C2766 | dehydrogenase/GMP reductase | 5’CAAAGACCGTTGATGTTGAGG3’ | 5’ATGAGGCAGATGGAATGAGAT3’. |


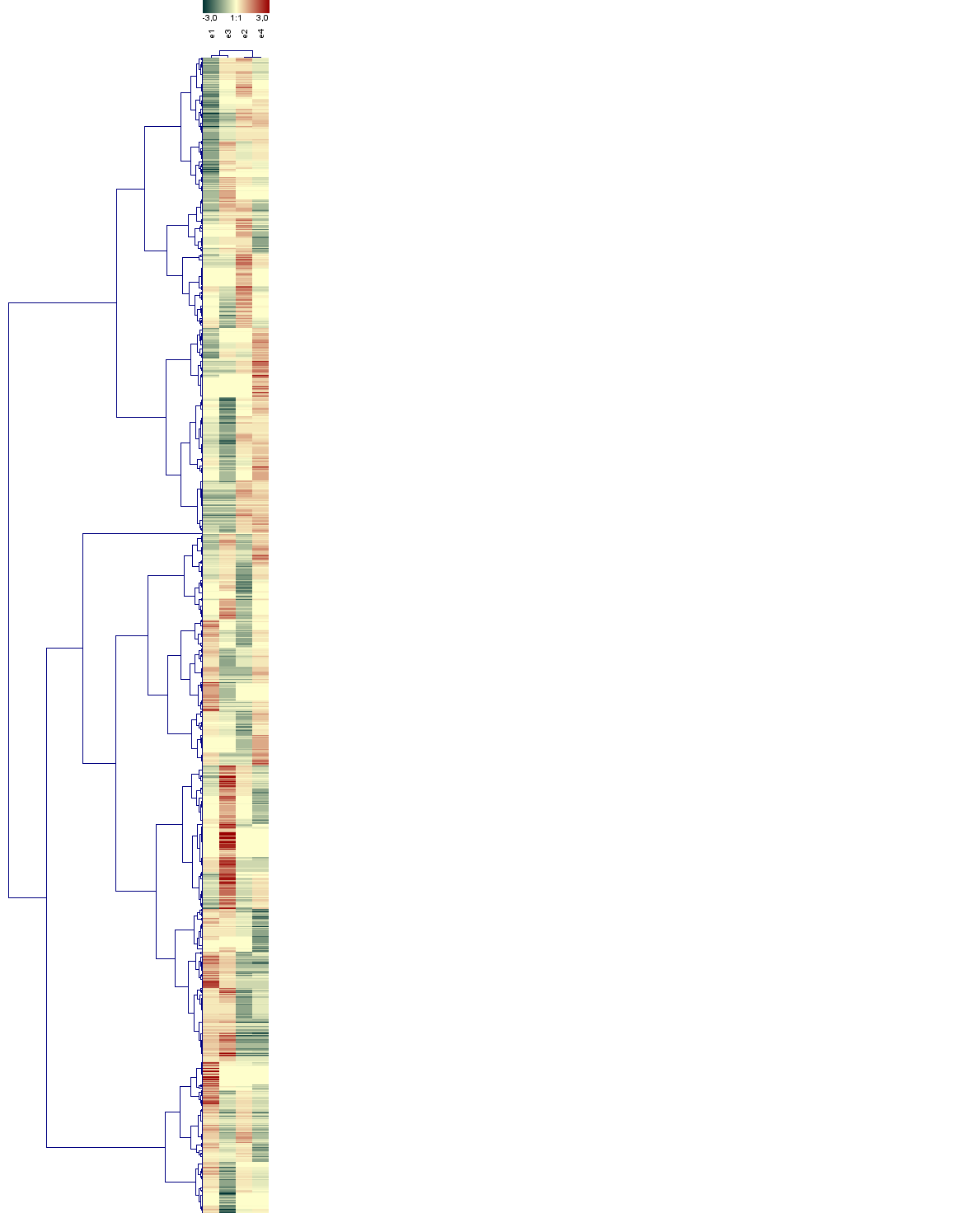


**Figure S1. The distribution of peach mesocarp derived ESTs under four different post-harvest conditions, using hierarchical clustering.**

1,402 Contigs with five or more ESTs were selected and normalized proportionally to the total number of ESTs in their corresponding contig (log 2). The EST frequency profile is displayed, indicating the points of significantly change between libraries. The green color indicates a decrease in the number of ESTs and red indicates an increase from the mean of each contig. The two-dimensional clustering is based on a Pearson`s correlation coefficient matrix. E1 = non-ripe; no long-term cold storage = R^-^, C^-^; E2 = ripe; no long-term cold storage = R^+^, C^-^; E3 = non-ripe; long-term cold storage = R^-^, C^+^; E4 = ripe: long-term cold storage = R^+^, C^+^.


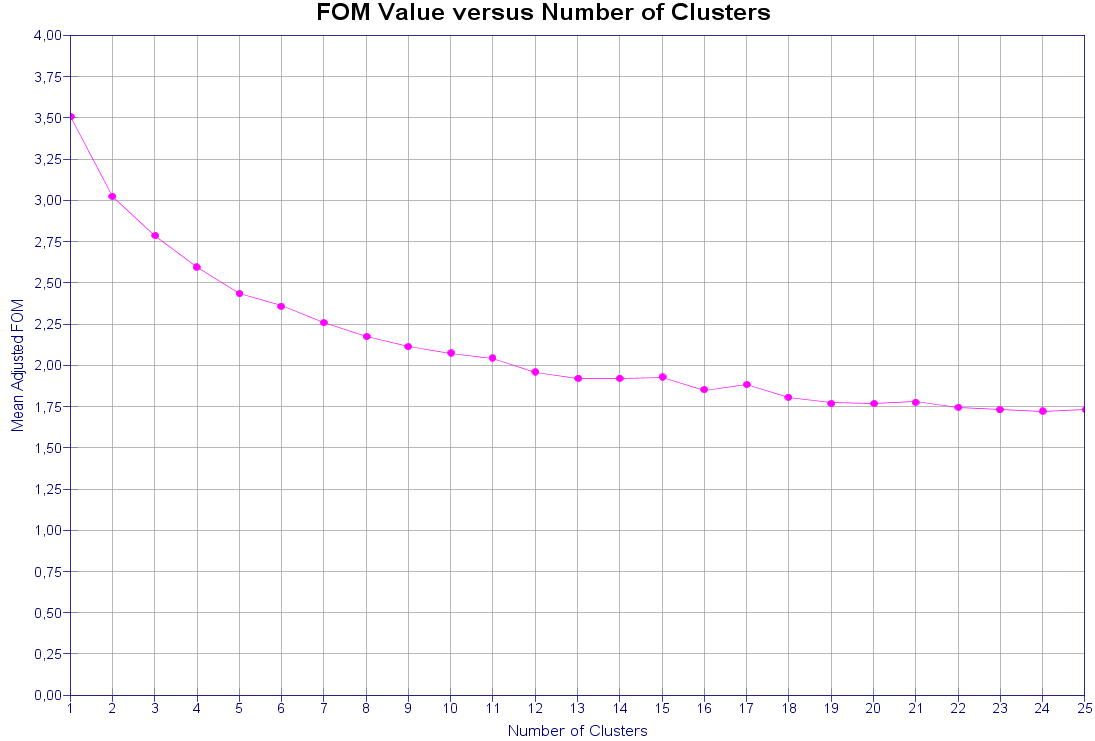


Figure S2. Figure of merit analysis. Figure of Merit (FOM) used to estimate the optimum number of clusters to use in the k-means method [49].
